# CoData on Patient Record Trajectory for Linkage (DataPRinT Linkage).

**DOI:** 10.23889/ijpds.v7i3.1782

**Published:** 2022-08-25

**Authors:** Sumeet Kalia, Michelle Greiver, Frank Sullivan, Conrad Pow, Aziz Sheikh

**Affiliations:** 1 University of Edinburgh; 2 University of St Andrews; 3 North York General Hospital

The linkage of Electronic Medical Records, Administrative and other data sources is highly valuable for research and health system monitoring. Once linked, combined resources can be analyzed to provide the answers to a variety of health questions that otherwise could not be answered. However, legislative and administrative barriers, including lengthy processes for data sharing agreements, may preclude timely linkage which is a key requirement during pandemics.

## Objectives

To develop a method using a patient’s health trajectory to probabilistically link primary care Electronic Medical Record (EMR) data with administrative and other data, without the need to transfer large datasets or identifiable information. To determine the legislative feasibility, accuracy and validity of this linkage process.

## Study Design

Identify data strings that do not directly identify patients and could be used as unique linkage variables. The data strings, which we are calling dataprints, are sufficiently similar over time in different databases. One example in Ontario, Canada, is the pattern of submitted health claims. For every patient seen by a family physician, there exists a unique pattern of dates/billing codes/diagnoses over time. These unique patterns are reasonably similar in EMR and administrative datasets. We will apply an algorithm which turns the string in the selected dataprints to an irreversibly hashed code for each person. The hashed code and no additional information will be provided by both data controllers to a trusted-third party who will determine which records match and send a mapping table to both. This enables analyses to be run in parallel, without divulging any direct person identifiers.

## Dataset

Individuals contained in the University of Toronto Practice Based Research Network (UTOPIAN). 

## Outcome Measures

Linkage quality will be assessed by the number of true matches and represented by sensitivity, specificity and positive and negative predictive values. 

## Results

The method will be evaluated against a validated, deterministically linked reference standard at North York General Hospital using de-identified EMR and hospital data. Results will inform processes to enable analyses across datasets while adhering to privacy legislation.

